# Supplementary material for: Content-rich biological network constructed by mining PubMed abstracts
Source: BMC Bioinformatics. 2004 Oct 8;5:147. doi: 10.1186/1471-2105-5-147 (PMC528731; doi:10.1186/1471-2105-5-147)
Supplement: Additional File 2 — The original results of the above study (non-essential files are deleted to keep the file size under the limit set by BMC bioinformatics). [file 1471-2105-5-147-S2.bz2 › chilibotAdditionalFile2/dip05/259ID8606778E1023/html/CDK2_TRXA.html]

 


 **CDK2** and **TRXA** 
  
Found 3 abstracts in PubMed, retrieved 3.  
 

 What does Google say? 
 PDF only 
| .edu only 

---

**Interactive relationship** (e.g. stimulation, inhibition, etc)

**Neutral relationship**- We report here the expression of a combinatorial library of constrained 20 residue peptides displayed by the active site loop of Escherichia coli thioredoxin  [ **TRXA** ] , and the use of a two hybrid system to select those that bind human  **Cdk2** .  Ref: 8606778 Nature, 1996

**Non-interactive relationship** (e.g. studied together, co-existance, homology, etc.)

- Abbreviations used ABD albumin binding domain of protein G APPI Alzheimer s amyloid beta protein precursor inhibitor BBP bilin binding protein BPTI bovine pancreatic trypsin inhibitor BSA bovine serum albumin CBD cellulose binding domain of cellobiohydrolase I CD circular dichroism  **Cdk2**  human cyclin dependent kinase 2  [ **CDK2** ]  CDR complementarity determining region CTLA 4 human cytotoxic T lymphocyte associated protein 4 FN3 fibronectin type III domain GSH glutathione GST glutathione S transferase hIL 6 human interleukin 6 HSA human serum albumin IC half maximal inhibitory concentration Ig immunoglobulin IMAC immobilized metal affinity chromatography K equilibrium constant of dissociation K equilibrium dissociation constant of enzyme inhibitor LACI D1 human lipoprotein associated coagulation inhibitor pIII gene III minor coat protein from filamentous bacteriophage f1 PCR polymerase chain reaction PDB Protein Data Bank PSTI human pancreatic secretory trypsin inhibitor RBP retinol binding protein SPR surface plasmon resonance  **TrxA**  E. coli thioredoxin  [ **TRXA** ]   Ref: 10931555 J Mol Recognit< MedlineTA>J Mol Recognit, 1996
